# Supplementary material for: Capsanthin Inhibits Atherosclerotic Plaque Formation and Vascular Inflammation in ApoE−/− Mice
Source: Biomedicines. 2022 Jul 23;10(8):1780. doi: 10.3390/biomedicines10081780 (PMC9332034; doi:10.3390/biomedicines10081780)
Supplement: Supplementary file 1 [file biomedicines-10-01780-s001.zip › biomedicines-1798484-supplementary.pptx]

## Slide 1
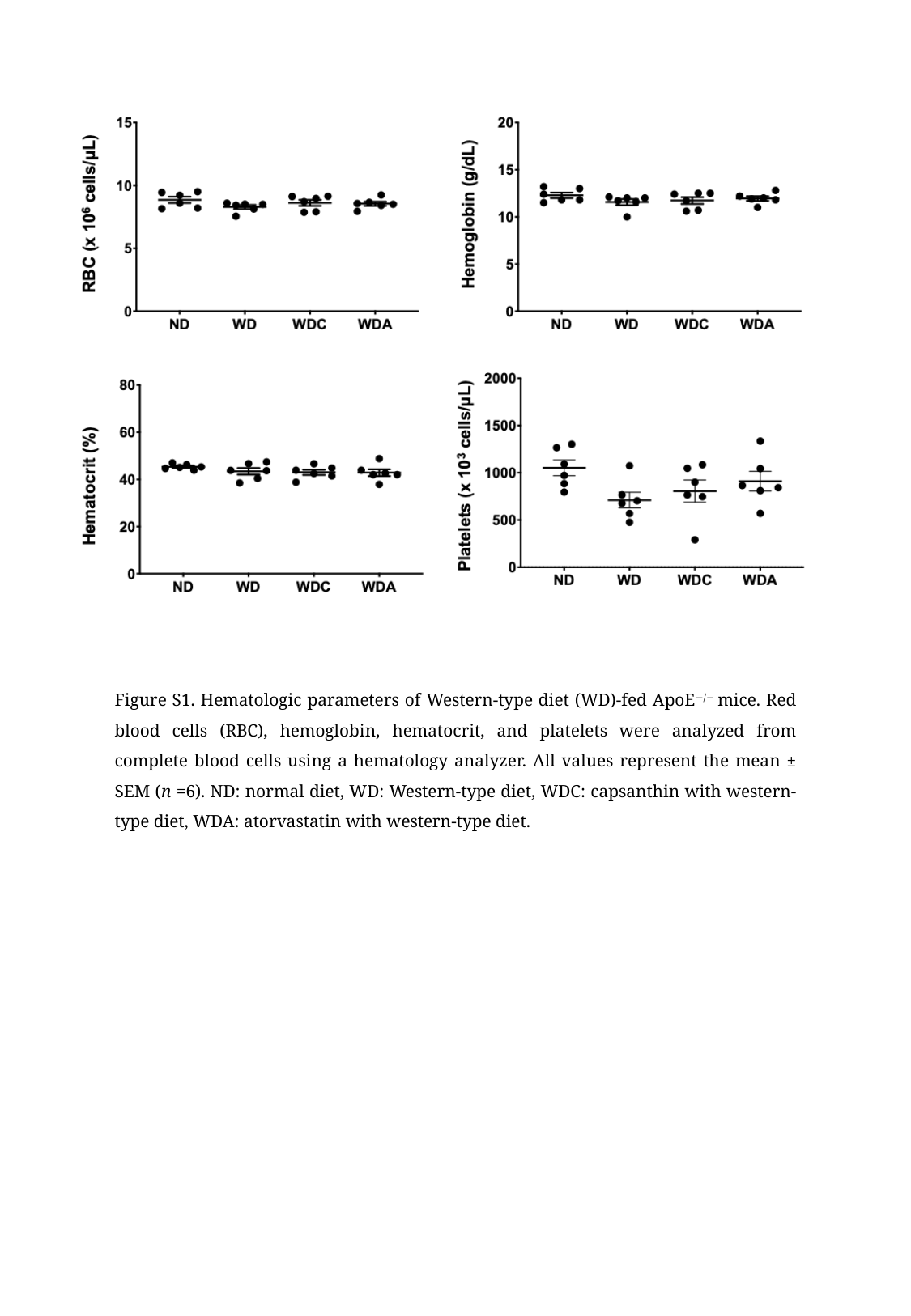

Figure S1. Hematologic parameters of Western-type diet (WD)-fed ApoE−/− mice. Red blood cells (RBC), hemoglobin, hematocrit, and platelets were analyzed from complete blood cells using a hematology analyzer. All values represent the mean ± SEM (n =6). ND: normal diet, WD: Western-type diet, WDC: capsanthin with western-type diet, WDA: atorvastatin with western-type diet.
